# Digitally Adapting LGBTQ-Affirmative Cognitive Behavioral Therapy for Chinese Men Who Have Sex With Men Living With HIV: User-Centered Design Approach

**DOI:** 10.2196/98541

**Published:** 2026-09-14

**Authors:** Weizi Wu, Xiaohan Song, John Pachankis, Mengyao Yi, Mengshu Li, Si Pan, Ying He, Fei Li, Yang Xiong, Xianhong Li

**Affiliations:** 1 Yale University School of Nursing, Orange, CT, United States; 2 School of Nursing, LKS Faculty of Medicine, The University of Hong Kong, Hong Kong, China (Hong Kong); 3 Xiangya School of Nursing, Central South University, 172 Tongzipo Road, Yuelu District, Changsha, Hunan, China, +86 18374961210; 4 Yale University School of Public Health, New Haven, CT, United States; 5 Second Xiangya Hospital of Central South University, Changsha, Hunan, China; 6 Kaifu District Psychiatric Clinic, Changsha, Hunan, China; 7 Xiangya Hospital Central South University, Changsha, Hunan, China

**Keywords:** men who have sex with men, HIV, cognitive behavioral therapy, digital health, mental health, minority stress

## Abstract

**Background:**

Chinese men who have sex with men living with HIV (MSMLWH) experience substantial psychological distress driven by minority stress and HIV-related challenges. However, culturally tailored digital mental health interventions that address HIV-specific maladaptive cognitive schemas and culturally specific psychosocial stressors remain scarce in China.

**Objective:**

This study aimed to systematically adapt an evidence-based cognitive behavioral therapy (CBT) intervention Effective Skills to Empower Effective Men (ESTEEM) into a WeChat (Tencent) Mini-Program–based intervention (iESTEEM) specifically for Chinese MSMLWH and to evaluate its preliminary feasibility and usability.

**Methods:**

We used a three-phase user-centered design approach guided by the Assessment, Decision, Adaptation, Production, Topical Experts, Integration, Training, and Testing (ADAPT-ITT) framework. The study proceeded in three phases: (1) a qualitative needs assessment using semistructured interviews with 20 MSMLWH (mean age 23.25, SD 3.08 years); (2) systematic intervention adaptation and platform development, including theater testing (n=5); and (3) a 2-week pilot study involving 10 MSMLWH and five counselors to evaluate feasibility, usability, and acceptability through focus groups and objective platform analytics.

**Results:**

Phase 1 identified 3 major themes of psychological distress: persistent health anxiety fueled by catastrophizing, intersectional stigma internalization, the disclosure dilemma, and intimacy barriers rooted in defectiveness and shame schemas. Participants also prioritized anonymity and bite-sized learning. Guided by these findings, iESTEEM was developed as a counselor-assisted, privacy-preserving WeChat Mini-Program incorporating HIV-specific scenarios, multimodal learning modules, and a back-end risk-alert system. During the 2-week pilot, participants logged into the platform 14.1 (SD 6.7) times per person and completed 134.3 (SD 103.1) minutes of learning activities; all participants accessed module 1, and 90% (9/10) accessed modules 2‐5. Anxiety scores decreased from 8.9 (SD 2.3) to 7.2 (SD 3.0), whereas depression scores remained stable. All participants expressed a willingness to continue using the program and to recommend it to peers. Participants and counselors endorsed its contextual relevance, privacy protections, and clinical utility.

**Conclusions:**

This study provides a theory- and evidence-informed model for culturally adapting digital mental health interventions for highly stigmatized populations. By integrating lesbian, gay, bisexual, transgender, and queer (LGBTQ)-affirmative CBT principles, HIV-specific adaptations, and a privacy-preserving, counselor-assisted WeChat Mini-Program, iESTEEM demonstrated promising preliminary feasibility, acceptability, and engagement among Chinese MSMLWH. These findings support the potential of culturally tailored digital interventions to expand access to psychological support for this stigmatized population in resource-constrained settings. Ongoing randomized controlled trials will further evaluate its efficacy, implementation outcomes, and mechanism of action.

## Introduction

While advances in antiretroviral therapy have substantially increased life expectancy, psychological distress remains a significant concern for men who have sex with men living with HIV (MSMLWH) [1-3]. In China, this vulnerability is further compounded by the intersections of sexual minority stress and HIV-related stress, including traditional expectations of filial piety and lineage continuity, concerns regarding HIV disclosure, and both enacted and internalized stigma associated with their sexual orientation and serostatus [4-6]. Consequently, Chinese MSMLWH experience disproportionately high rates of anxiety and depression, with prevalence estimates reaching up to 40% and 50.8%, respectively [7,8]. These mental health challenges are associated with poor treatment adherence, diminished quality of life, and adverse HIV-related health outcomes [9]. Despite the substantial psychological burden experienced by this population, accessible and culturally responsive mental health services for Chinese MSMLWH remain largely unmet [10,11].

Cognitive-behavioral therapy (CBT) is an established approach for reducing psychological distress among sexual minority populations; however, traditional face-to-face counseling is often constrained by high costs, limited mental health resources, and concerns regarding stigma and unintended disclosure [12-14]. Internet-based CBT (iCBT) has therefore emerged as a scalable, privacy-preserving alternative [15-17]. Recent mobile health (mHealth) interventions for MSMLWH in China have incorporated digital delivery, human-centered design, skills training, and coaching support to improve mental health and HIV-related outcomes and have demonstrated the feasibility and promise of digital mental health interventions in this population [18,19]. However, most largely focus on adjustment, coping, adherence, or emotional regulation and have paid limited attention to minority stress processes and HIV-specific maladaptive cognitive schemas.

Among existing interventions, the Effective Skills to Empower Effective Men (ESTEEM) program is a prominent, evidence-based lesbian, gay, bisexual, transgender, and queer (LGBTQ)—affirmative iCBT intervention specifically designed to reduce sexual minority stress among men who have sex with men (MSM) [20]. Through a structured 10-session protocol, ESTEEM promotes adaptive coping by normalizing minority stress experiences, restructuring maladaptive cognitive schemas, and reducing emotion-driven avoidance behaviors [21]. Clinical trials in the United States, cross-cultural adaptations in Romania, and our previous work among Chinese HIV-negative MSM have consistently demonstrated its effectiveness in improving mental health and reducing behavioral risks [15,22-24]. However, ESTEEM has primarily been evaluated among HIV-negative MSM and sexual minority stress [25,26], and it does not explicitly address HIV-related challenges such as health anxiety, disclosure dilemmas, and HIV-related stigma [27], particularly within the Chinese sociocultural context.

For Chinese MSMLWH, the coexistence of sexual minority stress and HIV-related stress creates a distinct dual-stress context that extends beyond the experiences of HIV-negative MSM [25,28]. Consequently, interventions designed primarily to address HIV-related adjustment or sexual minority stress alone may each address only part of the psychological burden experienced by this population. There is therefore a critical need to systematically adapt evidence-based interventions that integrate both perspectives and address the complex, culturally situated mental health needs of Chinese MSMLWH.

To bridge this gap, this study used the Assessment, Decision, Adaptation, Production, Topical experts, Integration, Training, and Testing (ADAPT-ITT) framework [29,30] to systematically adapt the evidence-based ESTEEM program into a WeChat (Tencent) Mini-Program (iESTEEM) for Chinese MSMLWH. Specifically, this study aimed to (1) qualitatively identify the psychological distress experiences, maladaptive cognitive schemas, and intervention needs of Chinese MSMLWH; (2) adapt the ESTEEM content and delivery platform to address these needs; and (3) preliminarily evaluate the feasibility and usability of iESTEEM. By integrating qualitative needs assessment with theory- and evidence-informed intervention adaptation, this study provides a systematic approach for digitally and culturally adapting an LGBTQ-affirmative CBT intervention to address minority stress and HIV-specific psychological challenges among Chinese MSMLWH.

## Methods

### Study Design and Setting

**Figure 1.**
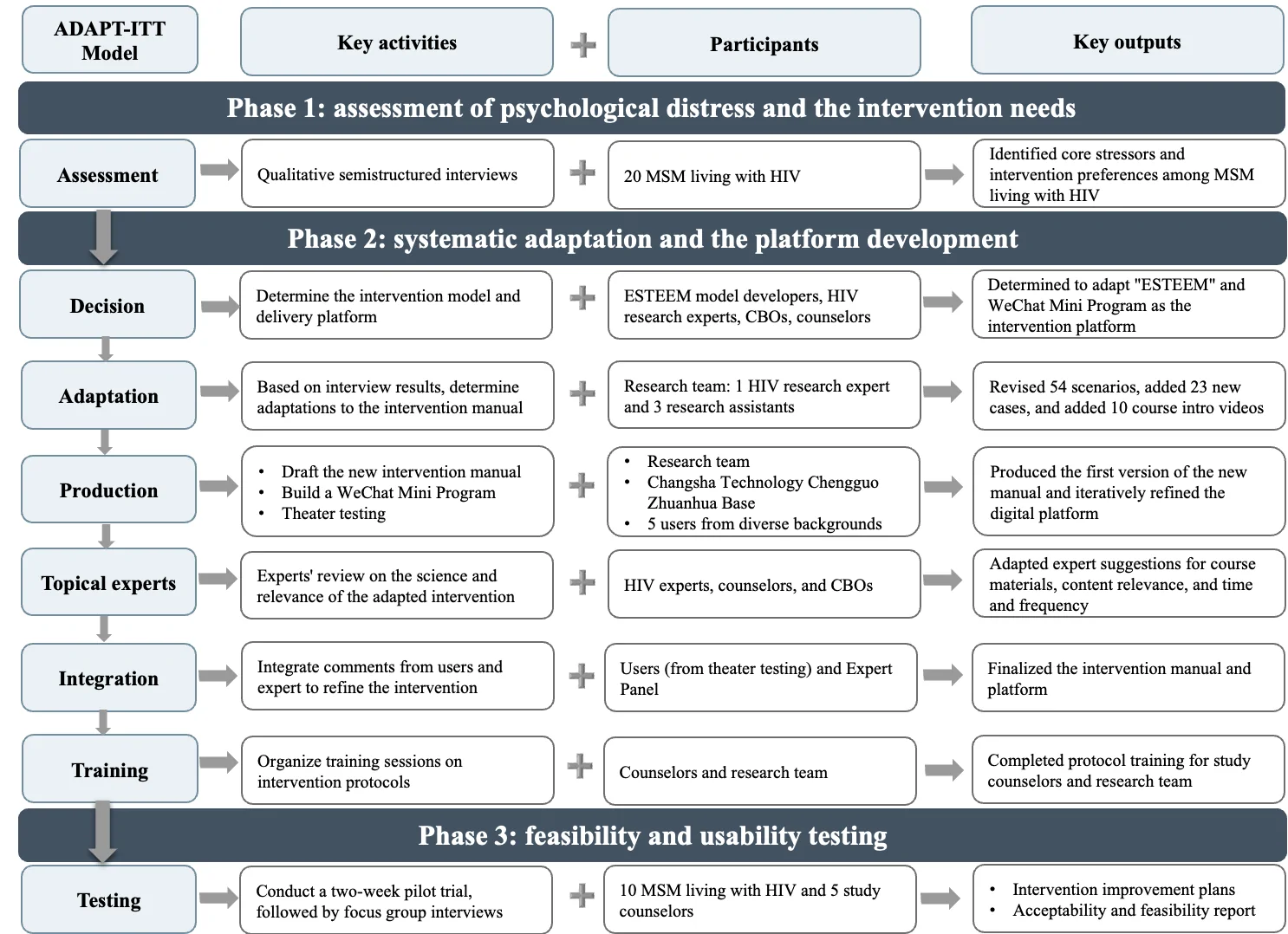
Overview of the systematic adaptation process using the ADAPT-ITT framework. The flowchart illustrates the study’s three-phase process: (1) needs and preference assessment, (2) systematic adaptation and platform development, and (3) feasibility and usability pilot testing. It highlights the eight steps of the ADAPT-ITT model, including assessment, decision, adaptation, production, topical experts, integration, training, and testing, as well as details the main activities, the involved stakeholders (MSM living with HIV, experts, and lesbian, gay, bisexual, transgender, and queer and other sexual orientations friendly community-based organizations), and the outputs generated at each stage for the adapted intervention on WeChat Mini-Program. ADAPT-ITT: Assessment, Decision, Adaptation, Production, Topical experts, Integration, Training, and Testing; CBO: community-based organization; MSM: men who have sex with men.

This study used a three-phase user-centered design approach guided by the eight-stage ADAPT-ITT framework (Figure 1). Phase 1 assessed psychological distress and intervention needs, phase 2 focused on intervention adaptation and WeChat Mini-Program development, and phase 3 involved pilot testing of feasibility, usability, and acceptability. Figure 1 presents the key activities, stakeholders involved, and outputs across all phases. This paper was prepared in accordance with CONSORT-EHEALTH (Consolidated Standards of Reporting Trials of Electronic and Mobile Health Applications and Online Telehealth) checklists and informed by Consolidated Criteria for Reporting Qualitative Research (COREQ).

The study was conducted in Hunan Province, China, with recruitment supported by the Changsha Zhongda Sunshine Social Work Service Center, a community-based organization that provides HIV testing, counseling, and sexual health services for sexual minority populations and people living with HIV.

### Phase 1: Assessment of Psychological Distress and Intervention Needs

#### Participants

Purposive sampling was used to ensure diversity in sociodemographic characteristics, clinical experience, and psychological distress, including varying levels of anxiety and depressive symptoms. Participants were eligible if they (1) were men aged ≥18 years, (2) self-identified as MSM, (3) had a confirmed HIV diagnosis, (4) had access to WeChat, and (5) provided informed consent. Individuals were excluded if they had hearing or language impairments or had participated in psychological treatments (eg, psychotherapy or support groups) or other intervention-based research within the previous three months.

#### Data Collection

Trained research assistants with prior experience in qualitative interviewing and CBT conducted semistructured, in-depth interviews lasting approximately 60‐90 minutes. The interview guide explored participants’ experiences related to HIV diagnosis, stigma and disclosure, social support, coping with sexual minority and HIV-related stress, treatment adherence, and preferences for the digital psychological intervention. The complete interview guide is provided in Table S1 in Multimedia Appendix 1.

Before the interview, participants completed standardized measures to characterize their baseline psychological symptom profiles. Anxiety was measured using the Generalized Anxiety Disorder 7-item (GAD-7) scale, in which scores >9 and >14 indicate moderate and severe anxiety, respectively (Cronbach α=0.90) [31]. Depression was measured using the Patient Health Questionnaire-9 (PHQ-9), with scores >9, >14, and >19 indicating moderate, moderately severe, and severe depression, respectively [32]. Sociodemographic information (eg, age, ethnicity, marital status, and education), behavioral, and clinical characteristics (eg, sexual behavior and substance use) were also collected.

#### Data Analysis

Audio recordings were transcribed verbatim and analyzed using thematic analysis supported by NVivo 11.0 (QSR International). Two researchers (WW and XL) independently reviewed and coded the transcripts following the six-step approach proposed by Braun and Clarke [33]. To ensure that qualitative findings directly informed intervention adaptation, the analysis followed a framework-guided thematic approach focused on 3 broad domains relevant to the ESTEEM intervention framework: psychological distress, underlying cognitive schemas, and behavioral patterns. Specific themes and subthemes emerged inductively through iterative coding and constant comparison and subsequently served as targets for intervention adaptation in phase 2. WW and XL (project principal investigator) met regularly throughout the analytic process to compare interpretations, refine the coding framework, and resolve discrepancies through consensus discussion. Recruitment and analysis proceeded concurrently, and no substantially new themes emerged in the final interviews, suggesting adequate thematic saturation for the study objectives. Descriptive statistics were used to summarize participants’ demographic, clinical, and psychological characteristics, including means, SDs, ranges, and frequency distributions. GAD-7 and PHQ-9 scores were categorized according to established cutoff values to describe symptom severity.

### Phase 2: Systematic Adaptation and Platform Development

Informed by the phase 1 assessment and the team’s prior studies [15,24], two decisions guided intervention development. First, ESTEEM was selected as the foundational intervention because of its demonstrated efficacy among Chinese HIV-negative MSM and prior cultural adaptation for the Chinese MSM population [24,34,35]. Second, the delivery platform was transitioned from a conventional web interface to a WeChat Mini Program to accommodate participants’ preferences for privacy, anonymity, and accessibility.

### Systematic Adaptation of the Intervention Manual

#### Overview

Content adaptation was guided by integrating phase 1 qualitative findings with the theoretical principles of LGBTQ-affirmative CBT underlying the ESTEEM framework. Recurrent HIV-related cognitive, emotional, and behavioral patterns were mapped to corresponding CBT strategies, including cognitive restructuring, cognitive reattribution, flexible thinking, communication rehearsal, and psychoeducation. A multidisciplinary research team iteratively adapted the original 10-session manual by identifying content to retain, delete, modify, or add, and subsequently developed a revised manual. Content modifications were documented in an adaptation matrix, and representative examples are summarized in Table S4 in Multimedia Appendix 2.

After the manual was revised, the adapted intervention was then developed into a WeChat Mini Program in collaboration with a technology company. The platform included dedicated interfaces for users, counselors, and administrators. The prototype underwent theater testing (n=5 participants) to optimize the user interface and content delivery flow. Finally, an expert panel comprising HIV researchers, counselors, and community-based organization (CBO) staff reviewed the intervention to assess its theoretical fidelity, cultural sensitivity, and clinical safety.

#### Format of Counselor Involvement

The iESTEEM was designed as a counselor-assisted intervention rather than a fully automated self-guided program. Participants completed the 10 intervention modules at a weekly cadence while receiving asynchronous counselor support integrated into each session [36]. Counselor involvement followed a standardized protocol with two primary functions: (1) skill reinforcement, whereby counselors reviewed cognitive restructuring assignments and provided individualized feedback to reinforce CBT skills; and (2) responsive support, whereby participants’ questions and concerns were addressed within 48 hours. This structure ensured ongoing therapeutic support while maintaining the flexibility of self-paced learning. Counselors were also responsible for responding to automated risk alerts generated by the platform and implementing the study safety protocol when indicated.

#### Training and Quality Assurance

To ensure intervention fidelity, all project counselors had prior experience working with sexual minority populations and possessed professional CBT certification, and all research assistants received standardized training before pilot implementation. Training covered the original ESTEEM model, HIV-specific adaptations, platform operation, and procedures for reviewing assignments, responding to participant inquiries, and managing the risk-alert system.

### Phase 3: Preliminary Feasibility and Usability Evaluation

#### Overview

The 2-week pilot was designed to evaluate preliminary feasibility and usability, including participants’ engagement with core intervention components and the operational feasibility of the counselor-assisted Mini-Program. The pilot involved 10 MSMLWH representing the spectrum of psychological distress identified in phase 1 and five project counselors. The 2-week duration was selected to allow participants to experience the complete intervention workflow and interact with all 10 modules. During the pilot, participants engaged with the platform’s core features, including module learning, skill practice, assignment completion, and asynchronous communication with counselors. Long-term adherence, sustained counselor workload, or implementation outcomes across the full 10-week intervention were beyond the scope of this pilot study.

After the 2-week pilot, separate focus group interviews (30‐60 minutes) were conducted with MSMLWH participants and counselors. The evaluation focused on three domains informed by previous implementation and digital mental health studies [37,38]: (1) perceived utility and contextual relevance, including perceptions of the intervention’s relevance to participants’ lived experiences and the usefulness of its therapeutic skills; (2) system usability, including barriers and facilitators to engagement, privacy protections, and workflow efficiency; and (3) implementation feasibility and acceptability, including therapeutic alliance, willingness to continue using and recommend the program, and suggestions for future optimization. The complete interview guide is provided in Table S2 in Multimedia Appendix 3. Feedback from participants and counselors was documented and used to identify areas for future optimization of intervention content, platform functionality, and implementation procedures prior to the ongoing randomized controlled trial.

#### Data Analysis

Feasibility and engagement were summarized descriptively using objective platform analytics, including login frequency, cumulative login duration, counselor-message interactions, learning duration, and module-level access. Continuous variables were summarized using means, SDs, medians, and ranges or IQRs, as appropriate. Given the small sample size and formative nature of the pilot, changes in GAD-7 and PHQ-9 scores were examined descriptively and visualized as exploratory symptom trajectories without inferential efficacy testing. Qualitative feedback from participants and counselors was summarized according to the predefined evaluation domains to identify areas for intervention and platform optimization.

#### Safety Monitoring and Risk Response

The Mini-Program incorporated a backend risk-alert system that automatically notified counselors when participants’ routine psychometric monitoring scores exceeded predefined clinical thresholds. Counselors were instructed to review the alert, contact the participant through the platform, conduct a brief risk assessment, and provide follow-up supports or referral as needed. Urgent safety concerns were escalated to the principal investigator and clinical team according to the study safety protocol.

#### Ethical Considerations

This study was conducted in accordance with the Declaration of Helsinki and approved by the Ethics Committee of Xiangya School of Nursing, Central South University (approval number 2023110901). All participants provided electronic informed consent through the secure iESTEEM Mini-Program and were informed that participation was voluntary and could be withdrawn at any time without penalty. Data were anonymized and stored on an encrypted, password-protected server. Participants received compensation of 50 RMB (US $7.43) for completing the phase 1 interviews and 50 RMB for completing the phase 3 pilot assessment.

## Results

### Phase 1: Assessment of Psychological Distress and Intervention Needs

#### Participant Profile

Participant characteristics are summarized in Table 1. Twenty MSMLWH participants were relatively young (mean 23.25, SD 3.08 years), predominantly highly educated (19/20, 95%), and had been living with HIV for 7.25 (SD 4.14) months since diagnosis. Baseline psychological screening revealed a substantial mental health burden, with 30% (6/20) of participants reporting moderate-to-severe anxiety symptoms and 60% (12/20) reporting moderate-to-severe depressive symptoms.

**Table 1.** Participant characteristics of phase 1 interview participants^a^.

Characteristics	Values (N=20)
Age (years), mean (SD)	23.25 (3.08)
Education level, n (%)	
Undergraduate	17 (85)
Graduate	2 (10)
Others	1 (5)
Employment status, n (%)	
Employed	7 (35)
Student	8 (40)
Others	5 (25)
Time since HIV diagnosis (months), mean (SD)	7.25 (4.14)
Time from diagnosis to ART^b^ initiation (months), mean (SD)	2.74 (3.20)
GAD-7^c^ score, mean (SD)	8.40 (3.90)
Moderate-to-severe anxiety (GAD-7≥10), n (%)	6 (30)
PHQ-9^d^ score, mean (SD)	10.90 (4.10)
Moderate-to-severe depression (PHQ-9≥10), n (%)	12 (60)

a Only key participant characteristics are displayed to summarize sample heterogeneity and psychological symptom burden; complete demographic and clinical data are available in Table S3 in Multimedia Appendix 4.

b ART: antiretroviral therapy.

c GAD-7: Generalized Anxiety Disorder 7-item.

d PHQ-9: Patient Health Questionnaire-9.

#### Psychological Distress

**Figure 2.**
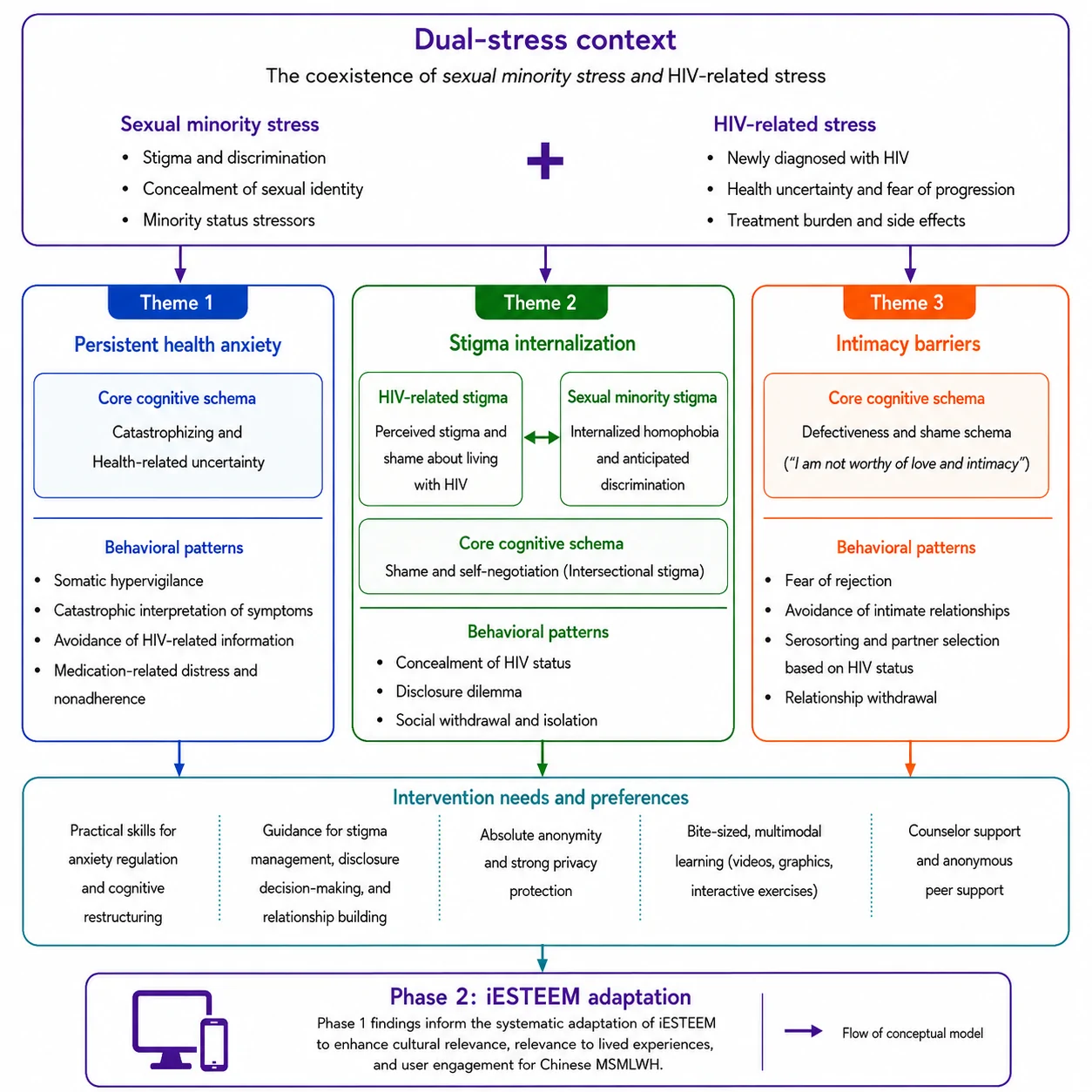
Conceptual model of psychological distress and intervention needs among Chinese men who have sex with men living with HIV (MSMLWH). The model illustrates how the dual-stress context of sexual minority stress and HIV-related stress contributes to three interconnected themes—persistent health anxiety, stigma internalization, and intimacy barriers—through underlying cognitive schemas and behavioral patterns. These findings informed intervention needs and preferences and guided the subsequent adaptation of iESTEEM. MSMLWH: men who have sex with men living with HIV.

The qualitative analysis identified 3 major themes of psychological distress, including persistent health anxiety, stigma internalization, and intimacy barriers (Themes 1‐3), as well as specific intervention needs and delivery preferences (Theme 4). Across themes, participants described how the coexistence of sexual minority stress and HIV-related stress generated maladaptive cognitive schemas that shaped emotional responses and coping behaviors. These findings informed the systematic adaptation of iESTEEM and are summarized in Figure 2.

### Theme 1: Persistent Health Anxiety Driven by Catastrophizing

Participants described a psychological trajectory characterized by persistent health anxiety following HIV diagnosis. Initial reactions often involved acute existential distress and catastrophic interpretations of HIV as an immediate threat to life. As the initial shock subsided, distress evolved into chronic health anxiety regarding disease progression, treatment effectiveness, and future uncertainty. This catastrophizing schema contributed to maladaptive coping behaviors, including hypervigilance toward bodily symptoms, avoidance behaviors of HIV-related information, and emotional distress associated with medication use.

*My depression really spiraled [post-diagnosis]. I used to feel like death was a million miles away. But now, taking those meds at night just forces me to face the fact that I’m (HIV) positive. And the side effects, the dizziness, the diarrhea, it’s like I can’t escape it from the second I open my eyes.*

[Participant A19]

### Theme 2: Intersectional Stigma Internalization and the Disclosure Dilemma

Participants described profound distress mainly arising from the intersection of HIV-related stigma and sexual minority stigma. Many perceived HIV-related stigma and moral judgments (eg, being viewed as “dirty” or “immoral”) as more psychologically harmful than stigma related to sexual orientation. External stigma frequently became internalized, leading participants to experience shame, self-disgust, and feelings of inferiority. Family relationships further complicated these experiences. In the context of filial piety and expectations of lineage continuity, participants often interpreted HIV diagnosis not merely as a medical condition but as a personal and moral failure that violated familial expectations. Consequently, they experienced a disclosure dilemma in which they simultaneously desired family support and understanding yet feared that disclosure would lead to disappointment, rejection, or emotional harm to their loved ones. This tension often led to concealment as a primary coping strategy, which protected them from anticipated judgment but also reinforced isolation and internalized shame.

*I honestly don’t know how to face my mom... she had such high expectations for me. Do I keep this from her for the rest of my life, or wait for the “right” time? If I lie forever, what happens if I eventually get full-blown AIDS and die? She’ll be old and gray by then. For a mother to have to bury her own child... keeping her in the dark until that very last moment just feels incredibly cruel. But telling her now? I don’t even know where to start. It feels like a lose-lose situation. I’m just completely torn on whether to disclose my status.*

[Participant A17]

### Theme 3: Intimacy Barriers and a Schema of Defectiveness and Shame

Participants reported that HIV diagnosis fundamentally altered their relational schemas and further expectations regarding intimate relationships. Although most participants understood the principle of “Undetectable = Untransmittable” (U=U), they often struggled to emotionally internalize this knowledge and continued to perceive themselves as dangerous, infectious, or damaged. This disconnect between intellectual understanding and emotional experience reflected a persistent schema of defectiveness and shame, whereby participants equated living with HIV with being fundamentally flawed or undesirable. Consequently, many anticipated rejection from HIV-negative partners, excessive guilt regarding potential transmission, and avoided intimate relationships. Serosorting (limiting partners to other HIV-positive individuals) frequently emerged as a strategy to minimize disclosure-related anxiety and perceived moral responsibility.

*If I fall for someone negative, persuading them to understand and accept me... I feel it is still quite difficult... I want to find a partner with whom I can share a life. Sometimes, I went to the hospital for medicine or a follow-up visit, I looked around a bit [for other MSMLWH] to see if there was anyone, maybe a potential partner.*

[Participant A01]

### Theme 4: Intervention Needs and Preferences

Beyond describing psychological distress, participants articulated specific requirements for the intervention design that directly informed the adaptation process. Participants prioritized practical coping skills over theoretical education and identified the period immediately following HIV diagnosis as the most critical window for support. To address anxiety, stigma, and relationship difficulties, they preferred actionable strategies delivered through highly private and easily accessible formats. Given concerns regarding unintended disclosure, participants strongly favored a WeChat Mini-Program and advocated for “bite-sized learning” (eg, sessions under 40 minutes) and multimodal learning experiences supplemented by counselor and peer support. These preferences provided the empirical basis for adapting both the intervention content and the WeChat Mini-Program delivery platform in phase 2.

### Phase 2: Systematic Adaptation and Platform Development

#### Content Adaptation Targeting Maladaptive Cognitive Schemas

**Figure 3.**
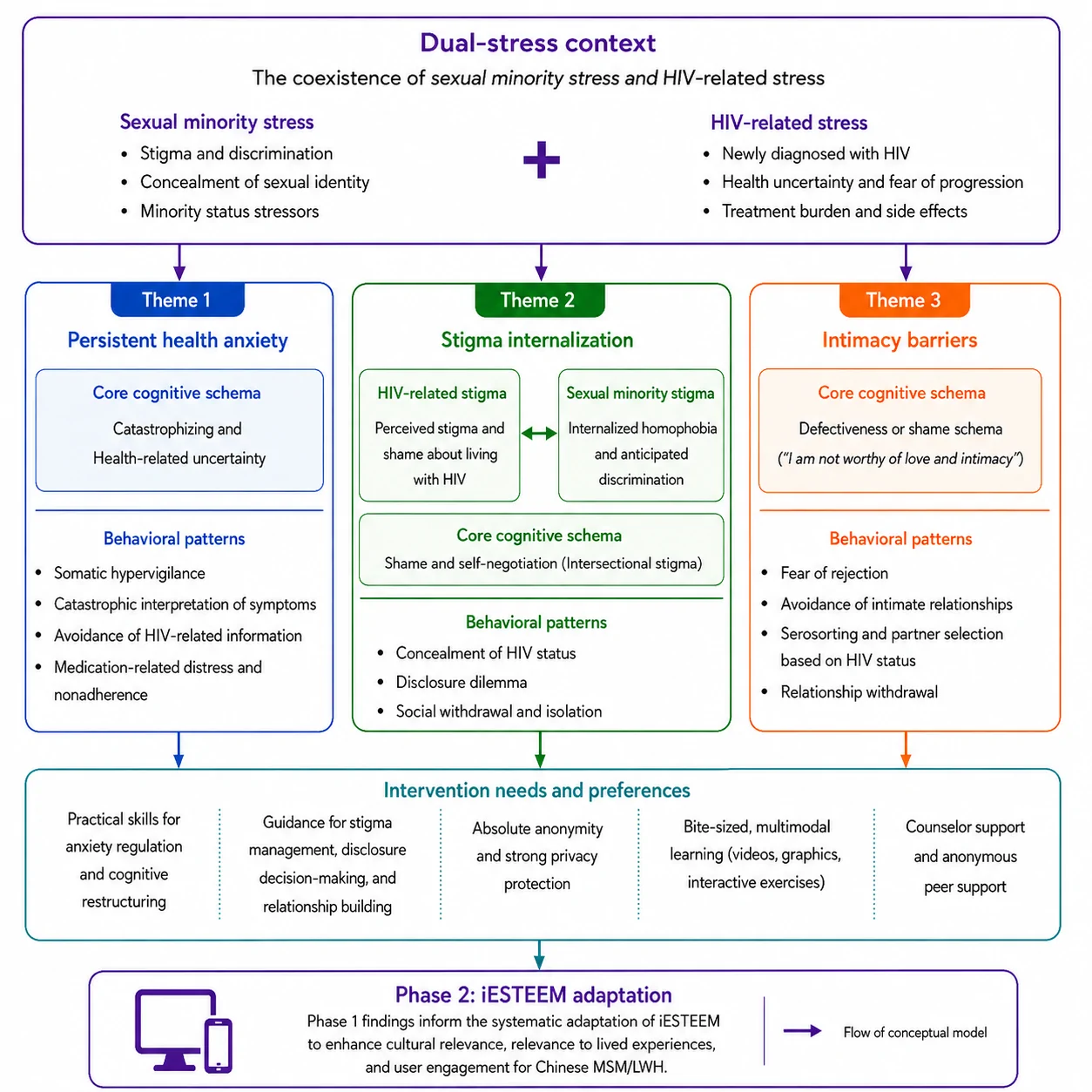
Phase 2 systematic adaptation and development of the iESTEEM workflow. The workflow illustrates the sequential process from identifying key needs and maladaptive schemas among Chinese men who have sex with men (MSM) living with HIV to selecting and tailoring cognitive behavioral therapy (CBT) principles, adapting intervention content, developing the WeChat Mini-Program, and iteratively refining the intervention through user feedback and expert consultation. CBT: cognitive behavioral therapy; MSM: men who have sex with men; MSMLWH: men who have sex with men living with HIV; U=U: undetectable equals untransmittable.

Guided by the phase 1 findings, the iESTEEM was systematically adapted and iteratively refined to address the dual-stress context and HIV-specific maladaptive cognitive schemas identified among Chinese MSMLWH (Figure 3; Table S4 in Multimedia Appendix 2). The adaptation broadened the original ESTEEM focus from solely addressing sexual minority stress to encompassing both sexual minority stress and HIV-related stress. Overall, 53 universal scenarios were retained, 54 scenarios were modified to incorporate HIV-specific content, and 23 new scenarios were created based on participant interviews.

Representative adaptations included four major domains. First, to address the disclosure dilemma associated with HIV stigma and filial piety, the generic “Coming Out” module was replaced with a “Dual Disclosure” scenario titled “Yezi’s Story,” which guides users to practice disclosing their dual identities (MSM and HIV status) and reinterpret parental shock and fear as temporary emotional reactions rather than inevitable rejection. Second, to address the health-related catastrophizing, cognitive reattribution exercises were added to challenge the tendency to interpret minor physical symptoms (eg, fatigue or dizziness) as signs of immune failure or mortality. Third, to address medication-related distress and nonadherence, goal-setting and cognitive reframing strategies were integrated to reposition medication-taking from a painful reminder of illness into a symbol of self-care and health protection. Fourth, to address intimacy barriers, the “U=U” principle was incorporated into core belief modification exercises, and new “Intimacy Dilemma” scenarios (eg, Mr Cao’s story) were developed to help users distinguish their HIV status from their value as romantic partners. The complete scenarios are provided in Table S4 in Multimedia Appendix 2.

#### Cultural Reframing of Intervention Brand

Building upon our previously adapted ESTEEM program for Chinese HIV-negative MSM (“Yi Si Tang”), the intervention for MSMLWH was renamed iESTEEM (Chinese: Ai Yi Si Tang) to reduce HIV-related stigma and enhance cultural relevance. The prefix “*i*” was intentionally selected for its phonetic resonance with “Ai (爱, meaning Love),” symbolizing self-love and empowerment while reinforcing the intervention’s emphasis on cognitive restructuring and positive identity reconstruction.

#### Delivery Adaptation of the iESTEEM Intervention

To address participants’ preferences for privacy, accessibility, and flexible learning identified in phase 1, iESTEEM was developed as a counselor-assisted, safety-monitored WeChat Mini-Program (Figure 3). The platform was designed to provide a highly private, user-friendly, and flexible digital environment that could seamlessly integrate into participants’ daily routines while minimizing concerns regarding unintended identity disclosure.

**Figure 4.**
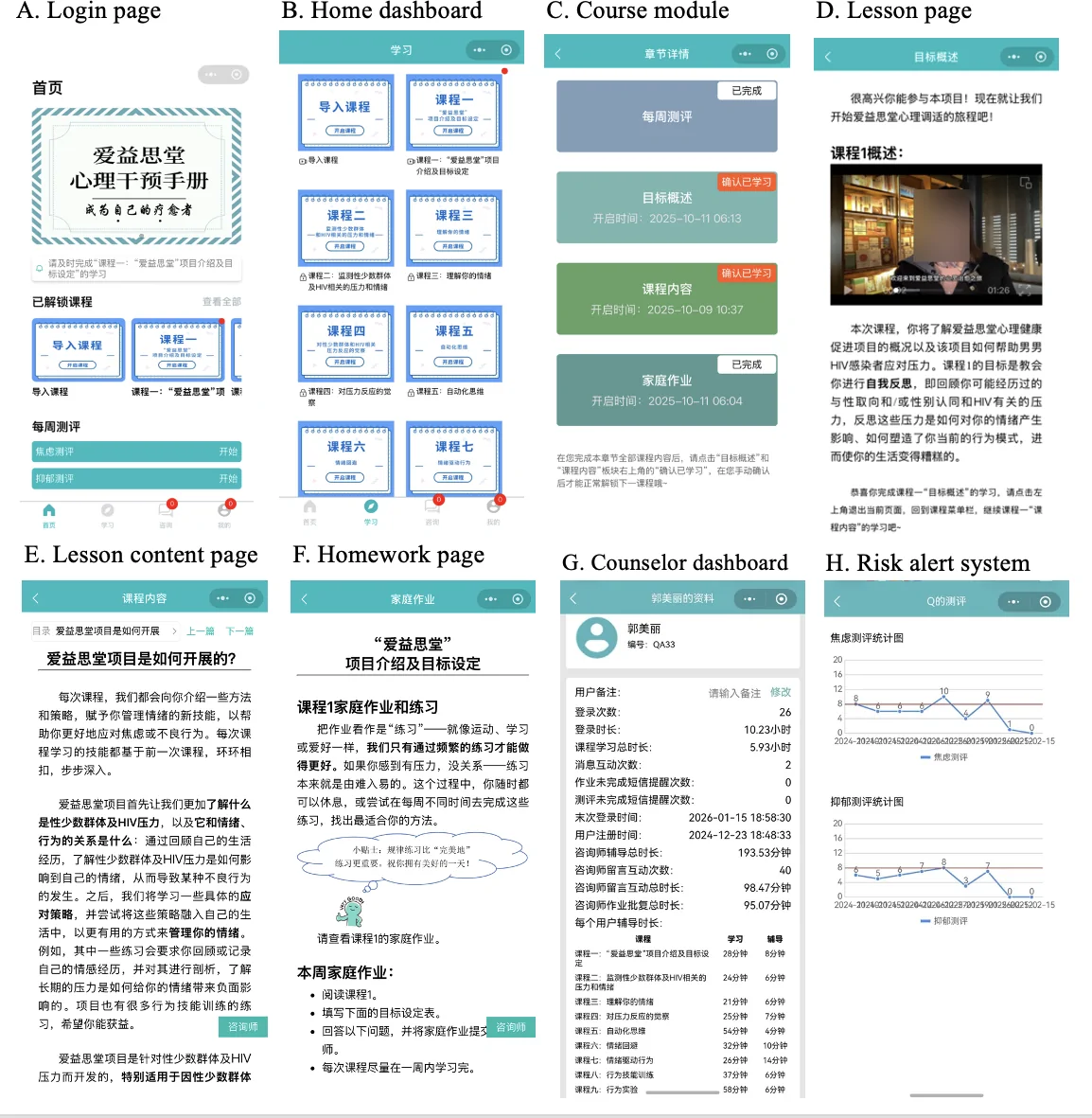
Digital architecture and functional modules of the iESTEEM WeChat Mini-Program. (A) Login page: the central hub featuring progress tracking, unlocked courses, and buttons for weekly psychometric monitoring (Anxiety and Depression scales). (B) Home dashboard: the linear, lock-step interface displaying the 10 core cognitive behavioral therapy (CBT) sessions plus one introductory session. (C) Course module: a structured single-session view requiring users to complete “Goal Setting,” “Course Content,” and “homework” sequentially. (D) Lesson page: an introductory video module designed to reduce cognitive load and enhance user interest. (E) Lesson content page: adapted CBT reading materials integrated with specific coping strategies. (F) Homework page: the interactive homework module featuring a “consult counselor” button (green icon) for asynchronous support. (G) Counselor dashboard: the centralized backend system granting counselors and research staff visibility into participant adherence. Key metrics include login frequency, cumulative study duration, and interaction logs to facilitate timely supervision. (H) Risk alert system: visual tracking of anxiety and depression trajectories over time. The red horizontal line indicates the preset clinical threshold; scores exceeding this line trigger the automated safety alert mechanism. Scores exceeding the predefined clinical threshold automatically generate counselor notifications and prompt follow-up safety assessment.

The front-end user interface consisted of 5 core modules: secure dual-verification login, structured course learning, assignment submission for skill consolidation, counselor messaging, and routine psychometric monitoring (Figures 4A-4F). To accommodate participants’ preferences for bite-sized, engaging learning, intervention content was delivered through multimodal materials, including short videos, graphics, and interactive case scenarios. This modular design enabled participants to engage with intervention content flexibly while maintaining structured progression through the 10-session program.

The back-end management system was designed to support monitoring, safety, and adherence (Figures 4G and 4H). The dashboard enabled administrators and counselors to monitor engagement indicators, including login duration, counseling duration, and assignment completion. An automated risk-alert system notified counselors when psychometric monitoring scores exceeded the predefined severity threshold and prompted follow-up safety assessments. Weekly reminders and asynchronous counselor messaging, with counselor responses provided within 48 hours, were implemented to promote adherence and ensure timely professional support.

#### Iterative Refinement and Optimization

The iESTEEM protocol underwent iterative refinement through theater testing and expert consultation to enhance usability, ecological validity, and cultural relevance. User feedback from theater testing informed several user-centered refinements, including simplification of the login process, increased font size, and strengthened engagement mechanism—the system was programmed to provide automated encouraging prompts and explicit reminders of counselor availability when users faced challenges, thereby aiming to enhance adherence. Expert feedback further informed adaptations that address culturally salient stressors, particularly marriage pressure and the family disclosure dilemma. The finalized intervention content and the technically optimized platform then proceeded to phase 3 to evaluate their feasibility and usability.

### Phase 3: Feasibility and Usability Testing

#### Study Participants

A total of 10 MSMLWH and 5 counselors participated in the 2-week pilot study and postintervention focus group interviews. As shown in Table S5 in Multimedia Appendix 5, these MSMLWH faced considerable psychological distress, with 60% (6/10) reporting moderate-to-severe anxiety and 70% (7/10) reporting moderate-to-severe depression.

#### Objective Feasibility and Engagement

Objective platform analytics demonstrated active and sustained engagement with the iESTEEM Mini-Program during the 2-week pilot period (Table 2). Participants logged into the platform 14.1 (SD 6.7) times per person and spent 3.83 (SD 2.25) hours per person on the platform, completed 134.3 (SD 103.1) minutes of learning activities per person, and engaged in 2.7 (SD 3.0) message interactions per person.

**Table 2.** Objective engagement metrics of the iESTEEM^a^ mini-program during the 2-week pilot (n=10).

Engagement metric	Mean (SD)	Median	Range (minimum–maximum)
Number of logins, n	14.1 (6.7)	14	4‐25
Total login duration (hours)	3.83 (2.25)	3.16	0.72‐7.50
Number of message interactions, n	2.7 (3.0)	2.5	0‐9
Total learning duration (minutes)	134.3 (103.1)	83.2	23.2‐331.2

a iESTEEM: adaptation of cognitive behavioral therapy intervention Effective Skills to Empower Effective Men into a WeChat mini-program–based intervention.

Engagement with individual modules was concentrated in the intervention’s introductory, psychoeducational, and cognitive components (Table 3). All participants accessed the introductory module, and 90% (9/10) accessed modules 2‐5. Engagement gradually declined in later behavioral modules, with 70% (7/10) accessing module 6 and 40% (4/10)‐50% (5/10) accessing modules 7‐10.

**Table 3.** Engagement with individual iESTEEM^a^ modules during the 2-week pilot (n=10).

Module	Content	Time spent (minutes), median (IQR)	Participants accessing module, n (%)^b^
1	Introduction and goal setting	25.3 (17.6‐51.1)	10 (100)
2	Monitoring sexual minority and HIV-related stress and emotions	20.3 (8.4‐22.8)	9 (90)
3	Understanding your emotions	10.1 (6.1‐22.5)	9 (90)
4	Awareness of stress responses	15.3 (5.1‐37.7)	9 (90)
5	Automatic thoughts	11.8 (5.9‐26.4)	9 (90)
6	Emotion avoidance	6.4 (1.2‐11.9)	7 (70)
7	Emotion-driven behaviors	2.6 (0.0‐8.2)	5 (50)
8	Behavioral skills training	0.0 (0.0‐8.5)	4 (40)
9	Behavioral experiments	0.0 (0.0‐5.6)	4 (40)
10	Relapse prevention	0.0 (0.0‐6.8)	4 (40)

a iESTEEM: adaptation of cognitive behavioral therapy intervention Effective Skills to Empower Effective Men into a WeChat mini-program–based intervention.

b Participants were considered to have accessed a module if the recorded time spent on that module was greater than 0 minutes.

#### Preliminary Symptom Trends

**Figure 5.**
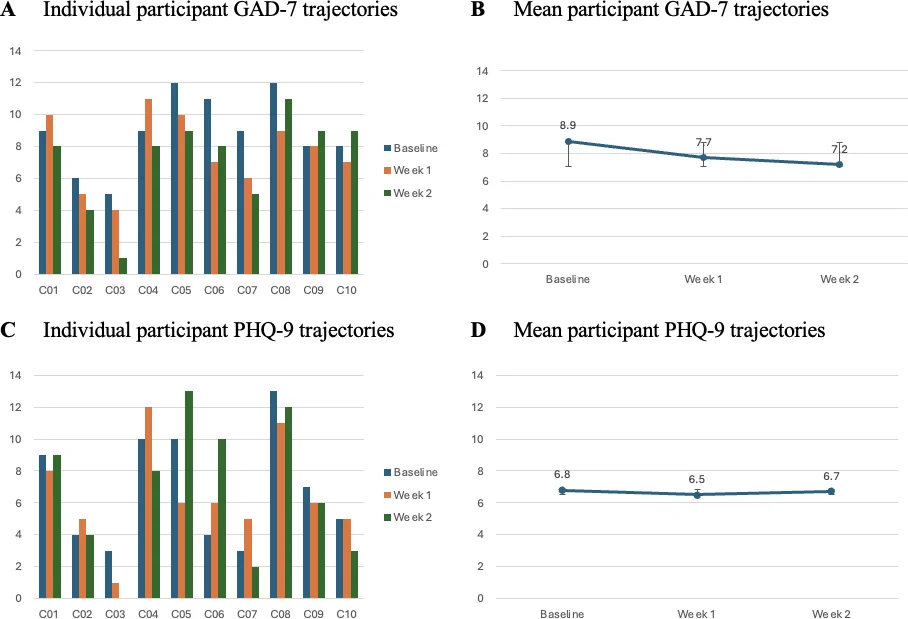
Preliminary anxiety and depression score changes during the 2-week pilot (n=10). (A) Individual participant Generalized Anxiety Disorder-7 (GAD-7) scores at baseline, week 1, and week 2. (B) Mean GAD-7 scores across the three assessment time points; error bars represent standard deviations. (C) Individual participant Patient Health Questionnaire-9 (PHQ-9) scores at baseline, week 1, and week 2. (D) Mean PHQ-9 scores across the three assessment time points; error bars represent standard deviations. Higher GAD-7 and PHQ-9 scores indicate greater severity of anxiety and depressive symptoms, respectively. The trajectories are presented descriptively to illustrate preliminary symptom patterns during the pilot and should not be interpreted as evidence of intervention efficacy. GAD-7: Generalized Anxiety Disorder 7-item; PHQ-9: Patient Health Questionnaire-9.

Although the pilot was not designed or powered to evaluate efficacy, descriptive trends suggested reductions in anxiety symptoms over the study period (Figure 5). The GAD-7 scores decreased from 8.9 (SD 2.3) at baseline to 7.2 (SD 3.0) at week 2. In contrast, the PHQ-9 scores remained relatively stable (baseline: mean 6.8, SD 3.5; week 2: mean 6.7, SD 4.4).

#### Perceived Utility and Acceptability

Participants and counselors endorsed the contextual relevance and perceived utility of iESTEEM. Participants reported that newly developed scenarios, particularly the “Dual Disclosure” and “Somatic Hypervigilance” exercises, closely reflected their lived experiences, describing them as “finally speaking our language” compared with generic HIV-related materials. Participants also valued the self-paced videos and cognitive exercises, which provided practical strategies for managing anxiety, stigma, and relationship concerns and were subsequently reinforced through individualized asynchronous counselor feedback.

#### Accessibility and Implementation Feasibility

The WeChat Mini-Program was perceived as highly accessible, private, and acceptable. Participants unanimously highlighted the advantages of the no-download, WeChat-embedded format. It reduced barriers to use and enabled an “invisible” mode of engagement that enhanced psychological safety by minimizing concerns regarding unintended disclosure, identity exposure, and HIV-related stigma. In addition, the bite-sized learning format facilitated integration of therapeutic activities into fragmented periods of daily life with minimal disruption to work and social routines.

Counselors emphasized the clinical utility of the backend management system. The dashboard enabled real-time monitoring of engagement metrics and automated identification of participants experiencing elevated psychological distress, facilitating timely follow-up and individualized support through the integrated risk-alert system. Counselors particularly valued the contextual cross-referencing functions, which allowed seamless navigation between participant assignments and relevant intervention modules and substantially improved the efficiency and precision of asynchronous counseling.

#### Overall Acceptability

Overall satisfaction was high. All participants expressed willingness to continue using the platform and stated they would recommend iESTEEM to peers. One technical challenge involving delayed counselor notifications was identified during the pilot. However, participants reported that the high quality of the therapeutic alliance and the sincere “human touch” of the counselor’s detailed responses largely offset the negative impact of the notification delays. Identification of this issue directly informed subsequent technical refinement for the future full-scale trial.

## Discussion

### Principal Findings

To our knowledge, this study is among the first to systematically adapt and pilot test an LGBTQ-affirmative, counselor-assisted digital iCBT intervention specifically for Chinese MSMLWH. Three principal findings emerged. First, our findings suggest that psychological distress among Chinese MSMLWH is shaped by a distinct dual-stress context in which sexual minority stress and HIV-related stress interact to produce HIV-specific maladaptive cognitive schemas and intervention needs. Second, this study demonstrates a theory- and evidence-informed approach for digitally and culturally adapting an existing affirmative CBT intervention through qualitative assessment and the ADAPT-ITT framework. Third, our findings from the pilot study support the preliminary feasibility and acceptability of delivering privacy-preserving, safety-monitored CBT through the WeChat Mini-Program with asynchronous counselor support.

Our findings suggest that psychological distress among Chinese MSMLWH is shaped by the intersection of sexual minority stress and HIV-related stress, extending Meyer minority stress model [39] within the Chinese context. Many participants perceived HIV-related stigma as even more threatening than sexual minority stigma. An HIV diagnosis acted as a “magnifier” of existing sexual minority stress [40], fueling an internalization process characterized by intersectional shame and a diminished sense of self-worth. Importantly, this internalized stigma extended beyond emotional distress to compromise health-promoting behaviors, particularly medication adherence. Distinct from the unintentional nonadherence commonly observed in chronic disease management, many participants engaged in intentional nonadherence, with high rates of missed doses across both study phases (18/20, 90% in phase 1 and 10/10, 100% in phase 3). For these individuals, taking medication symbolized a stigmatized and damaged identity. Consequently, some skipped doses to temporarily reclaim a sense of normalcy and avoid confronting their HIV status. This aligns with previous studies showing that stigma is a key barrier to medication adherence and psychological care [41,42]. Collectively, these findings suggest that intervention for MSMLWH should address internalized stigma and identity reconstruction alongside symptom management. By fostering cognitive flexibility and self-acceptance, the iESTEEM program seeks to support the development of a healthier, more integrated sense of self [43].

These findings further delineate HIV-related anxiety as a complex spectrum characterized by maladaptive cognitive schemas, particularly catastrophizing and somatic hypervigilance. Participants frequently interpreted minor physical sensations as signs of disease progression, thereby maintaining persistent vigilance and anxiety. This finding is consistent with previous research demonstrating that HIV-related stress often manifests somatically, whereby anxiety amplifies awareness and misinterpretation of benign physical symptoms [44]. Similar cognitive distortions were reflected in participants’ responses to the “U=U” principle [45,46]. Although most participants understood U=U intellectually, this knowledge was insufficient to overcome the fear of transmitting HIV to a partner, and often resulted in intimacy avoidance [47]. Collectively, these findings suggest that HIV-related distress among MSMLWH is maintained not by a lack of knowledge but by discrepancies between scientific knowledge and emotionally held beliefs rooted in internalized stigma. Therefore, interventions for MSMLWH should move beyond HIV education and directly target catastrophizing, emotional reasoning, and negative self-perception through a schema-focused and identity-affirming CBT approach.

Interpretation of the observed “social support paradox” requires contextualizing family relationships within the Chinese social-cultural context [48]. Given the participants’ young age and the absence of legal same-sex marriage, support systems often relied heavily on natal families rather than intimate partners. The family relationships in this context, traditionally a stress buffer [49], paradoxically become a primary source of distress, unlike the protective role frequently described in Western studies of “chosen families” [50,51]. Consequently, many participants engaged in a complex disclosure cost-benefit analysis, in which the perceived risks of disclosure outweighed the anticipated benefits of family support [52]. These findings highlight the potential value of alternative sources for social support. Consistent with previous research [53], many participants viewed peer support as a potentially safe and affirming space for sharing experiences that could not be openly discussed with family members. Although iESTEEM does not currently include peer-support functions, future digital interventions may benefit from incorporating secure, moderated peer-to-peer support components. Such platforms could leverage the “helper-therapy principle,” in which transitioning from a passive help-seeker to an altruistic peer supporter facilitates identity reconstruction [54]. At the same time, iESTEEM seeks to strengthen existing family support systems by helping participants navigate disclosure decisions and improve communication with family members.

Finally, our findings highlight the importance of user-centered and need-informed digital intervention design [17,55]. The evolution of iESTEEM from a web-based platform to a WeChat Mini-Program was driven not only by consideration of accessibility and convenience but also by participants’ need for privacy and identity protection. Unlike standalone mobile apps with visible icons, the WeChat-embedded format provided an “invisible” mode of engagement that reduced concerns regarding unintended disclosure and HIV-related stigma [56]. Participants also strongly preferred bite-sized, multimodal learning materials over traditional text-heavy content. This preference is consistent with cognitive load theory, whereby anxiety and emotional distress may reduce working memory capacity, making lengthy textual material difficult to process [57]. Delivering intervention content through brief videos and animated scenarios may therefore facilitate engagement and information retention among emotionally distressed populations [58,59]. Together, these findings support a user-centered, culturally localized approach that integrates the unique digital ecosystem of China with the psychosocial needs of Chinese MSMLWH and may provide a promising model for improving access to mental health care among stigmatized populations [60,61].

### Limitations

The study has several limitations. First, the representativeness and generalizability of the findings may be limited. The qualitative assessment (N=20) and pilot phases (n=10) involved small, geographically restricted samples recruited from Hunan Province. Participants were also relatively young, highly educated, and recruited through a community-based organization, which may have introduced selection bias toward individuals who were more digitally engaged and more willing to seek psychological support. Future studies should evaluate the intervention in larger, more diverse populations and examine potential subgroup differences, such as age, digital access, and clinical complexity, in intervention uptake and effectiveness.

Second, the study was designed as a formative adaptation and feasibility study and did not include a control group, limiting causal inference regarding the preliminary symptom improvements observed during the pilot. Consequently, we were unable to determine the relative contributions of specific intervention components, including self-directed learning modules, psychoeducational content, and asynchronous counselor support, to the observed changes [43,62]. In addition, the pilot focused on preliminary feasibility and usability and did not comprehensively evaluate 10-week implementation outcomes, such as full intervention completion, counselor engagement and workload, or longer-term engagement patterns and adherence challenges. Future studies should evaluate these implementation outcomes and intervention mechanisms in larger, more diverse samples, and an ongoing randomized controlled trial is examining the efficacy of iESTEEM for mental health and behavioral outcomes.

### Conclusion

This study systematically adapted the evidence-based ESTEEM program into iESTEEM, a culturally tailored, LGBTQ-affirmative, counselor-assisted WeChat Mini-Program designed specifically for Chinese MSMLWH. Our findings suggest that the intersection of sexual minority stress and HIV-related stress gives rise to distinct HIV-specific maladaptive cognitive schemas and intervention needs that are insufficiently addressed by existing interventions. By integrating qualitative assessment, the ADAPT-ITT framework, and user-centered digital design, this study provides a theory- and evidence-informed model for digitally and culturally adapting mental health interventions for highly stigmatized populations. The preliminary acceptability and feasibility observed during the pilot study support the promise of privacy-preserving, safety-monitored digital interventions for improving access to psychological support among Chinese MSMLWH. The ongoing randomized controlled trial will further evaluate the efficacy, implementation outcomes, and mechanisms of iESTEEM in improving mental health and health-related behaviors in this population.

## Supplementary material

10.2196/98541 Multimedia Appendix 1 Semistructured interview guide for needs and preferences assessment among MSMLWH (Phase 1).

10.2196/98541 Multimedia Appendix 2 Systematic adaptation of the iESTEEM intervention manual.

10.2196/98541 Multimedia Appendix 3 Interview guide for post pilot focus group interviews (Phase 3).

10.2196/98541 Multimedia Appendix 4 Demographic and clinical characteristics of HIV-Positive MSM interview participants (N=20).

10.2196/98541 Multimedia Appendix 5 Clinical and behavioral characteristics of participants in feasibility and usability testing (N=10).

## Abbreviations

ADAPT-ITT: Assessment, Decision, Adaptation, Production, Topical experts, Integration, Training, and Testing
CBO: community-based organization
CBT: cognitive behavioral therapy
CONSORT-EHEALTH: Consolidated Standards of Reporting Trials of Electronic and Mobile Health Applications and Online Telehealth
COREQ: Consolidated Criteria for Reporting Qualitative Research
ESTEEM: Effective skills to empower effective men
GAD-7: Generalized Anxiety Disorder 7-item scale
iCBT: internet-based CBT
iESTEEM: adaptation of cognitive behavioral therapy intervention Effective Skills to Empower Effective Men into a WeChat mini-program–based intervention
LGBTQ: Lesbian, gay, bisexual, transgender, and queer
mHealth: mobile health
MSM: men who have sex with men
MSMLWH: men who have sex with men living with HIV
PHQ-9: Patient Health Questionnaire-9
U=U: Undetectable=Untransmittable
