# Supplementary material for: Digitally Adapting LGBTQ-Affirmative Cognitive Behavioral Therapy for Chinese Men Who Have Sex With Men Living With HIV: User-Centered Design Approach
Source: J Med Internet Res. 2026 Sep 14;28:e98541. doi: 10.2196/98541 (PMC13573822; doi:10.2196/98541)
Supplement: Supplementary file 1 [file jmir-v28-e98541-s002.docx]

**Table S4. Systematic Adaptation of the *i*ESTEEM Intervention Manual**

| **Adaptation** | **Key Elements Adjusted** | **Representative Content Examples** |
| --- | --- | --- |
| **Retention** | CBT Core Framework & Sexual Minority Content | - Retained the structure and logic of the original 10-session CBT modular curriculum. - Retained 53 original case scenarios specifically addressing sexual minority stress (e.g., coming out, internalized homophobia). |
| **Deletion** | Irrelevant Behavioral Scenarios | - Removed stress scenarios applicable only to HIV-negative individuals, such as "Negotiating PrEP use" (Pre-exposure Prophylaxis) or "Anxiety regarding HIV testing," as these are not relevant to the diagnosed population. |
| **Modification** | 1. Conceptual Expansion: From Single to Dual Pressure | - Expanded Intervention Goal: Shifted focus from solely "Sexual Minority Stress" to addressing "Dual Pressure" (Sexual Minority Stress + HIV-related Stress). - Added Definition of HIV-related Stress: Defined as stress stemming from disease uncertainty, treatment side effects, public stigma, and the self-stigma/inferiority developed after internalization. Highlighted its impact on depression, anxiety, concealment behaviors, medication adherence, and quality of life. |
|  | 2. Scenario Adaptation: Integrating Intersectional Stigma | - **Example 1 (Family Rejection):**   **Original**: Parents rejected Mr. Ya's gay identity.  **Adapted**: Parents now reject his HIV status even more vehemently. They call him "dirty" and say he "got what he deserved" (Retribution). Whenever he visits home, they enforce isolation (e.g., requiring separate utensils), causing Mr. Ya profound grief and anger.   - **Example 2 (Internalized Stigma):**   **Original**: Mr. De felt inferior growing up as a gay man.  **Adapted**: After the HIV diagnosis, his self-loathing intensified. He now interprets the infection as "karmic retribution" for his sexual orientation, believing he is fundamentally flawed. |
|  | 3. Skill Training Revisions: Contextualizing CBT Techniques | - **ARC (Antecedent-Response-Consequence) Exercise**: The trigger event was modified to "A medication reminder alarm ringing during a company dinner," prompting end-users to analyze the conflict between adherence and fear of identity exposure. - **Flexible Thinking**: The core scenario was adjusted to "Deciding whether to disclose HIV status to parents" or "Informing a sexual partner of HIV status." - **Automatic Thoughts**: Added HIV-specific negative cognitions, such as "Internalized fear of AIDS," "Social withdrawal due to contagion fear," and "Anxiety that career prospects are ruined by mandatory health checks." |
|  | 4. Detailed Case Revision: Cognitive Restructuring (The "Yezi" Story) | **Scenario Background:**  "Yezi" faced a conflict between Filial Piety and authenticity. "Yezi" used to be close to his parents but drifted away to hide his HIV status, trapping himself in a web of lies. He feared disclosure (Gay + HIV) would destroy their relationship.  **Applied "Flexible Thinking" (Cognitive Restructuring):**  1. **Identify Catastrophic Thought:** "If I tell them, the family bond is broken forever."  2. **Generate Alternative Interpretations:**  - *Alternative A: "My parents love me; they care about my health and would want to know my true status. Sharing this secret might actually relieve the distance between us."*  *- Alternative B: "Disclosing might cause emotional waves, but it doesn't mean I will lose their love and support. They might be shocked initially due to lack of knowledge or fear, not because they hate me. If I can explain and educate them, their prejudice can change."*  **Outcome:** After generating these alternatives, Yezi realized that disclosure is a process, not a sudden disaster, and empowered to attempt disclosure to gain family support. |
| **Addition** | 1. Empowerment Principles & Scientific Knowledge | - **Reconstructing Self-esteem**: Introduced the core belief that " individuals living with HIV have equal rights to sex and love" to counter self-stigma. - **Psychoeducation:** Incorporated the "U=U" (Undetectable = Untransmittable) principle to alleviate transmission anxiety. |
|  | - 2. HIV-Specific Behavioral Strategies: Added 23 new scenarios targeting HIV stressors. | **New Exercise 1:** Medication Adherence as "Self-Care Ritual"   - Step 5 (Goal Setting): "I aim to maintain strict daily adherence." - Step 4 (Barrier Analysis): Identifying triggers.   External: "Meds interrupt work/play."  Internal: "Fear of identity exposure (e.g., colleagues seeing pills)."   - Step 3 (Catastrophic Thinking): Identifying the fear. "If people know I have HIV, I will lose my job and friends, bring shame to my family, and live in shadows forever." - Step 2 (Core Belief Identification): Pinpointing the root schema. "HIV infection is disgraceful/shameful." - Step 1 (Cognitive Reframing): Shift the meaning of pill-taking from "a painful reminder of being a patient" to "a proactive ritual of self-love and agency." Taking meds is an act of fighting for one's future, not submitting to the disease.   **New Scenario A:** Intimacy & Self-Worth Reconstruction   - Mr. Cao bravely disclosed his status to his partner and was deeply moved by the acceptance. However, plagued by deep inferiority, he vowed to cherish this relationship at all costs. Over time, even when his partner made unreasonable demands, Mr. Cao felt unable to refuse. He would think, "I don't deserve to say no to such a 'good' partner who accepts my status." He is even terrified to ask about his partner's health status, fearing it might shatter the relationship. Trapped in this dynamic, he no longer feels support or joy but doesn't know how to escape. - **Core Belief Identified:** "I am damaged goods; anyone who loves me is doing me a favor." - **Intervention (Core Belief Modification):**   - Challenge: Is your value defined solely by the virus?  - Reframing U=U: "U=U means I am medically safe. I bring love, care, and value to this relationship equal to my partner."  Goal: Empower users to set boundaries and pursue equal, healthy intimacy.  **New Scenario B:** Future Outlook & Career   - Mr. Wu, currently job hunting, once envisioned a bright future. However, the diagnosis plunged him into despair. Convinced he will fail the mandatory pre-employment health checks for his dream jobs, he now views his path as "grey and hopeless." He has abandoned all personal aspirations, buying a home, a car, or striving for success. His only plan is to wait until his parents pass away, then retreat to a small town to live out the rest of his life in isolation. - **Cognitive Distortion Identified:** "Fortune Telling" (predicting a negative future without evidence) and "Overgeneralization" ("If I can't be a civil servant, I can't do *any* job"). - **Intervention (Problem Solving & Cognitive Restructuring):**   - Reality Testing: Guide Mr. Wu to distinguish between actual structural barriers (e.g., civil service bans) and perceived total exclusion (e.g., private sector opportunities).  - Action Plan: Shift focus from "what I have lost" to "what is still possible." Set small, value-based career goals to regain a sense of agency.  Goal: Move from "passive resignation" to "active life planning" within realistic constraints.  **New Scenario C:** Health Anxiety & Somatic Hypervigilance   - Mr. Pu woke up with a mild rash and slight fatigue. - **Automatic Negative Thought (Catastrophizing):** "The medication isn't working. My immune system is failing. I am progressing to AIDS." - **Maladaptive Behavior:** Spent 3 hours checking symptoms online (Cyberchondria) and feeling panic. - **Intervention (Cognitive Reattribution):**   - Examine the Evidence: "I have been adherent to meds. My last viral load was undetectable."  - Reattribute Cause: "The rash is likely a mosquito bite or allergy (benign cause), and fatigue is from staying up late."  Goal: Break the cycle of interpreting benign physical sensations as signs of mortality. |
|  | Content Presentation Format | Added 10 new introductory videos to help end-users quickly grasp the main content of each session. |
