# Supplementary material for: Digitally Adapting LGBTQ-Affirmative Cognitive Behavioral Therapy for Chinese Men Who Have Sex With Men Living With HIV: User-Centered Design Approach
Source: J Med Internet Res. 2026 Sep 14;28:e98541. doi: 10.2196/98541 (PMC13573822; doi:10.2196/98541)
Supplement: Supplementary file 2 [file jmir-v28-e98541-s004.docx]

**Table S3. Demographic and Clinical Characteristics of HIV-Positive MSM Interview Participants (N=20)**

| Characteristics | Category | M ± SD |
| --- | --- | --- |
| Age (years) | — | 23.25 ± 3.08 |
| Date of Diagnosis (Month-Year) | — | 05.2022 - 02.2023 |
| Time from Diagnosis to ART (months) | — | 2.74 ± 3.20 |
| Characteristics | Category | n (%) |
| Ethnicity | Han Chinese | 18 (90.0) |
|  | Other | 2 (10.0) |
| Educational background | High school or lower | 1 (5.0) |
|  | Undergraduate | 17 (85.0) |
|  | Graduate | 2 (10.0) |
| Relationship Status | Significant others | 7 (35.0) |
|  | Single | 13 (65.0) |
| Monthly Income (CNY) | < 3000 | 8 (40.0) |
|  | 3000 ~ 5000 | 7 (35.0) |
|  | 5000 ~ 8000 | 5 (25.0) |
| Employment Status | Employed | 7 (35.0) |
|  | Student | 8 (40.0) |
|  | Temporary employed | 3 (15.0) |
|  | Unemployed | 2 (10.0) |
| Sexual Identity | Homosexual | 16 (80.0) |
|  | Bisexual | 4 (20.0) |
| Smoking | Yes | 1 (5.0) |
|  | No | 19 (95.0) |
| Alcohol Consumption | Yes | 4 (20.0) |
|  | Quitted | 2 (10.0) |
|  | No | 14 (70.0) |
| Daily Medication Frequency | Once daily | 15 (75.0) |
|  | Twice daily | 4 (20.0) |
|  | Missing | 1 (5.0) |
| Missed Doses (Past Month) | Yes | 18 (90.0) |
|  | No | 1 (5.0) |
|  | Missing | 1 (5.0) |
| Frequency of Missed Doses | Mean ± SD | 2.44 ± 1.51 |
| Psychological Support Received (Past 3 Months) | Yes | 8 (40.0) |
|  | No | 12 (60.0) |
| Counseling/Therapy Frequency (Past 3 Months) | Once a week | 1 (5.0) |
|  | Once a month | 2 (10.0) |
|  | Once every few months | 8 (40.0) |
|  | Several times a year | 9 (45.0) |
| Sexual Behavior (Past 3 Months) | Category | n (%) |
| Number of sexual partners | 0 | 3 (15.0) |
|  | 1 | 9 (45.0) |
|  | >1 | 8 (40.0) |
| Among those with multiple partners (n=8) | All fixed partners | 2 (25.0) |
|  | All casual partners | 3 (37.5) |
|  | Both fixed and casual | 3 (37.5) |
| Condomless Anal Intercourse | 0 times | 13 (65.0) |
|  | 1 time | 3 (15.0) |
|  | ≥ 3 times | 4 (20.0) |
| Condomless Oral Intercourse | 0 times | 12 (60.0) |
|  | 1 time | 4 (20.0) |
|  | 2-5 times | 3 (15.0) |
|  | >5 times | 1 (5.0) |
| Knew partner's HIV status | Yes | 12 (60.0) |
|  | No | 8 (40.0) |
| Disclosed HIV status to partner | Yes | 13 (65.0) |
|  | No | 7 (35.0) |
| Intoxicated during sex (Alcohol) | Yes | 3 (15.0) |
|  | No | 17 (85.0) |
| Substance use during sex (Rush, Marijuana, etc.) | Yes | 15 (75.0) |
|  | No | 5 (25.0) |
| Mental Health Status (Past 2 Weeks) | Category | n (%) |
| Anxiety (GAD-7) | Minimal | 4 (20.0) |
|  | Mild | 10 (50.0) |
|  | Moderate | 4 (20.0) |
|  | Severe | 2 (10.0) |
| Depression (PHQ-9) | None | 0 (0.0) |
|  | Mild | 8 (40.0) |
|  | Moderate | 8 (40.0) |
|  | Moderately severe | 3 (15.0) |
|  | Severe | 1 (5.0) |

Note. ART. Antiretroviral Therapy. GAD-7. Generalized Anxiety Disorder 7-item. PHQ-9. Patient Health Questionnaire 9-item. The total score for the GAD-7 ranges from 0 to 21. Severity is classified as: minimal (0-4), mild (5-9), moderate (10-14), and severe (15-21). The PHQ-9 total score ranges from 0 to 27. Severity is categorized as: none (0-4), mild (5-9), moderate (10-14), moderately severe (15-19), and severe (20-27).
