# Supplementary material for: Digitally Adapting LGBTQ-Affirmative Cognitive Behavioral Therapy for Chinese Men Who Have Sex With Men Living With HIV: User-Centered Design Approach
Source: J Med Internet Res. 2026 Sep 14;28:e98541. doi: 10.2196/98541 (PMC13573822; doi:10.2196/98541)
Supplement: Supplementary file 3 [file jmir-v28-e98541-s005.docx]

**Table S5. Clinical and Behavioral Characteristics of Participants in Feasibility and Usability Testing (N=10)**

| **Variables** | **Category** | **Frequency**  **(n)** | **Percentage**  **(%)** |
| --- | --- | --- | --- |
| Daily Dosing Frequency | Once daily | 6 | 60.0 |
|  | Twice daily | 4 | 40.0 |
| Medication non-adherence  (Past 1 Month) | Yes | 10 | 100 |
|  | No | 0 | 0 |
| Frequency of Missed Doses  (Per Month) | 2 times | 3 | 30.0 |
|  | 3 times | 1 | 10.0 |
|  | ≥ 4 times | 6 | 60.0 |
| Received Psychological Support  (Past 3 Months) | Yes | 4 | 40.0 |
|  | No | 6 | 60.0 |
| Frequency of Counseling  (Past 3 Months) | Monthly (Once a month) | 1 | 10.0 |
|  | Sporadic (Several times/year) | 3 | 30.0 |
| **Mental Health Status (Past 2 Weeks)** | **Category** | **Frequency**  **(n)** | **Percentage**  **(%)** |
| Anxiety (GAD-7) | Minimal | 2 | 20.0 |
|  | Mild | 2 | 20.0 |
|  | Moderate | 4 | 40.0 |
|  | Severe | 2 | 20.0 |
| Depression (PHQ-9) | None | 0 | 0 |
|  | Mild | 3 | 30.0 |
|  | Moderate | 2 | 20.0 |
|  | Moderately Severe | 4 | 40.0 |
|  | Severe | 1 | 10.0 |

Note. GAD-7. Generalized Anxiety Disorder 7-item. PHQ-9. Patient Health Questionnaire 9-item. The total score for the GAD-7 ranges from 0 to 21. Severity is classified as: minimal (0-4), mild (5-9), moderate (10-14), and severe (15-21). The PHQ-9 total score ranges from 0 to 27. Severity is categorized as: none (0-4), mild (5-9), moderate (10-14), moderately severe (15-19), and severe (20-27).
