# Supplementary material for: Digitally Adapting LGBTQ-Affirmative Cognitive Behavioral Therapy for Chinese Men Who Have Sex With Men Living With HIV: User-Centered Design Approach
Source: J Med Internet Res. 2026 Sep 14;28:e98541. doi: 10.2196/98541 (PMC13573822; doi:10.2196/98541)
Supplement: Supplementary file 4 [file jmir-v28-e98541-s001.doc]

**Table S1. Semi-structured Interview Guide for Needs and Preferences Assessment Among Men Who Have Sex with Men Living with HIV (Phase 1).**

| **Interview Theme** | **Sample Questions & Probes** |
| --- | --- |
| Diagnosis reaction | - **Initial Reaction:** Could you describe the circumstances surrounding your HIV diagnosis? What was your immediate emotional reaction and thought process upon receiving the result? What were your first actions? - **Perception of HIV:** What does "living with HIV" mean to you personally? How has this diagnosis altered your self-perception? How do you perceive the association between HIV and the MSM community? Did you hold any specific views about people living with HIV before your diagnosis, and have these views changed? |
| Disclosure & Social support | - **Disclosure Experience:** Have you disclosed your HIV status to anyone?   *(If yes)* How did you decide whom to tell and when? How did they react, and how did their reaction impact you? Do they also know about your sexual orientation?  *(If no)* What are your primary concerns or barriers regarding disclosure?   - **Peer Dynamics:** Do you have close friends within your social network who are also living with HIV? Have you discussed HIV-related topics with them? |
| Biopsychosocial Impact | - **Daily Functioning:** How has the diagnosis impacted your daily life, specifically regarding work, study, sleep, and diet? - **Psychological Impact:** Have you experienced any significant changes in your emotional state or personality (e.g., anxiety, depression, irritability)? - **Behavioral Changes:** Have there been changes in your sexual behaviors (e.g., frequency, partner selection, disclosure to partners)? Have you engaged in any substance use (e.g., poppers/rush, meth) or self-harm behaviors? - **Stress Sources:** What are your current primary sources of stress? Do you feel that the HIV diagnosis has exacerbated the stress associated with your sexual minority identity (Dual Pressure)? |
| Coping & Treatment Adherence | - **Coping Strategies:** How do you currently cope with or alleviate this stress? Do you find these methods effective, and how sustainable are they? - **Treatment Perceptions:** What are your thoughts on Antiretroviral Therapy (ART)? Did you initiate treatment immediately, and what factors influenced that decision? - **Adherence Barriers:** Have you ever missed doses? If so, what were the reasons? Do you perceive a link between your emotional state and your medication adherence (e.g., intentional non-adherence due to stigma)? |
| Preference for iESTEEM Implementation | - **Willingness:** If an online psychological intervention like this were available, would you be willing to participate? Why or why not? - **Program Format:** Are you willing to commit to a 10-session consecutive online self-guided curriculum (approx. 1 hour/session)? - **Privacy & Confidentiality:** Do you have any specific concerns regarding data security and privacy protection in the context of online counseling? - **Expectations of Counselors:** What qualities or characteristics do you look for in a counselor? What specific expectations do you have regarding the counselor's role and interaction style? - **Needs:** Which specific aspects of your well-being or mental health do you hope to improve through this intervention? - **Additional Recommendations:** Do you have any further suggestions to facilitate the successful implementation and delivery of this program? |
