# Supplementary material for: Digitally Adapting LGBTQ-Affirmative Cognitive Behavioral Therapy for Chinese Men Who Have Sex With Men Living With HIV: User-Centered Design Approach
Source: J Med Internet Res. 2026 Sep 14;28:e98541. doi: 10.2196/98541 (PMC13573822; doi:10.2196/98541)
Supplement: Supplementary file 5 [file jmir-v28-e98541-s003.doc]

**Table S2. Interview Guide for Post-Pilot Focus Group Interviews (Phase 3)**

| **Interview Theme** | **Sample Questions & Probes** |
| --- | --- |
| **System Usability & User Experience** | - **Navigation & Logic**: How would you describe your experience navigating the WeChat Mini-Program? Did the operational flow feel intuitive? - **Interface Design**: How do you perceive the visual design (UI) and layout? Was the text readable and the multimedia content (videos/audio) playable without lag? - **Technical Barriers**: Did you encounter any technical glitches or system errors during the two weeks (e.g., issues with notifications or loading)? |
| **Acceptability of Intervention Content** | - **Cultural Resonance**: Did the case scenarios and examples presented in the course resonate with your personal experiences as an HIV-positive MSM? Were there any parts that felt "foreign" or irrelevant? - **Content Utility**: Which specific modules or skills (e.g., cognitive restructuring, relaxation) did you find most useful? Why? - **Information Load**: How did you find the volume of reading material and the difficulty level of the homework? Was it too demanding? |
| **Feasibility of Implementation Mode** | - **Platform Preference:** Compared to traditional apps or websites, what are the advantages or disadvantages of using the WeChat Mini-Program for this intervention? - **"Fragmented Learning":** How did the "short burst" format (introductory videos + brief readings) fit into your daily routine? Did it facilitate your engagement? - **Privacy Perception:** Did the dual-verification system and the Mini-Program format make you feel secure regarding your privacy? |
| **Adherence & Engagement Barriers** | - **Barriers to Completion:** *(For those with lower adherence)* What were the primary factors that hindered you from logging in or completing sessions? (Probe: Time constraints, emotional avoidance, technical issues?) - **Facilitators:** *(For those with high adherence)* What motivated you to persist with the program? |
| **Therapeutic Alliance (Counselor Role)** | - **Perceived Support:** How would you describe your interaction with the study counselor? Did you feel "heard" and understood despite the asynchronous nature of the communication? - **Feedback Mechanism:** Was the counselor's feedback on your homework helpful? Did you receive the feedback in a timely manner? |
| **Future Recommendations** | - **Optimization:** If we were to roll this out to a larger group, what is the one thing we absolutely must change or improve? - **Sustainability:** Would you recommend iESTEEM to your friends in the community? Why or why not? |
